# Cardiovascular Functioning Features in Individuals with Connective Tissue Dysplasia Engaged in Sports for the Disabled

**DOI:** 10.3390/sports14020069

**Published:** 2026-02-05

**Authors:** Kamiliia Vinokurova, Anna Zakharova, Yulia Zinovieva, Arseniy Epifanov, Anna Galdobina, Ekaterina Sharkova, Felix Blyakhman

**Affiliations:** 1 Institute of Physical Education, Sports and Youth Policy, Ural Federal University, Ekaterinburg 620002, Russia; sport_tsp@mail.ru (A.Z.); namastespace@mail.ru (A.E.); lady.taranchuk@mail.ru (A.G.); sharkkat8@yandex.ru (E.S.); feliks.blyakhman@urfu.ru (F.B.); 2 Department of Functional Diagnostics, Sverdlovsk Regional Hospital No. 2, Ekaterinburg 620000, Russia; zya@obl2.ru; 3 Department of Biomedical Physics, Ural State Medical University, Ekaterinburg 620028, Russia

**Keywords:** disabilities, adaptive sports, physical exercises, cardiorespiratory system, LV false tendons, LV electromechanical function, heart functional reserve, cardiac adaptation to physical load

## Abstract

Objectives: Connective tissue dysplasia (CTD) is associated with disorders of collagen synthesis and is widely spread among the healthy population and people with disabilities. In the heart, primarily in the left ventricle (LV), CTD manifests itself as the formation of false tendons (LVFTs) to maintain close-to-normal LV pump function. This exploratory work is devoted to the search for general patterns of cardiac response to physical activity in athletes with disabilities, CTD, and LVFTs. The extent to which “the type of sports or the type of disability” determines the involvement of the heart’s functional reserve is the main testable question of the proposed research. Methods: The group under this study included 610 individuals with disabilities aged from 6 to 60 years with at least two transverse and/or oblique FTs per LV. Participants represented different sports disciplines (n = 10) and various forms of disabilities (n = 4). Cardiovascular indicators were obtained by means of standard TTE, impedance cardiography for hemodynamic monitoring in active orthotest, resting, and stress 12-lead ECG. Exercise testing of the athletes was performed with the use of appropriate methods of physical loading. In total, 141 parameters of cardiorespiratory function and exercise performance per participant were recorded. Statistical analysis of the dataset obtained across sports types or disability types was performed using one-way ANOVA or the Kruskal-Wallis test, depending on the assumptions of normality and homogeneity of variance. Results: Most importantly, it was found that only maximum relative oxygen consumption (VO_2_max, mL·kg^−1^·min^−1^) as a reliable indicator of the heart’s functional reserve and the corrected QT (QTc, ms) interval as an integral measure of the heart’s electrical activity demonstrated statistically significant differences across the sports specialization or the disability type. In particular, significance values (P) for VO_2_max across athletic disciplines and nosology categories were equal to 0.00063 and 0.01028 (one-way ANOVA), while for QTc they were 0.00001 and 0.02185 (Kruskal-Wallis), respectively. Furthermore, the type of disability had a lower impact on VO_2_max and QTc than the type of athletic activity. Conclusions: In athletes with disabilities and CTD, sport specialization may involve the heart’s functional reserve to a greater extent than the type of disability. To prescribe training loads for people with disabilities and CTD, individual cardiology screening with an emphasis on VO_2_max and QTc is necessary.

## 1. Introduction

According to reports of the WHO [1], over one billion individuals worldwide have significant disabilities, and the global prevalence of people with disabilities is increasing substantially. Moreover, rehabilitation and abilitation of people with disabilities is not limited to medical support but should also demonstrate a systematic approach in various areas of life, including sports and physical education. These forms of activity promote compensation for missing functions and health limitations, and are aimed at developing potential capabilities for further social adaptation. From this point of view, adaptive sport is one of the most effective tools for social integration in active life and the participation of individuals with disabilities in society.

The implementation of specific guidelines on adequate and safe training prescriptions and exercise dosing in individuals with disabilities is critically necessary. This would promote appropriate matching of functional capabilities and existing limitations in these people’s physiology-based choice of exercise activities aimed at both health prevention and partial compensation of current health impairments. To do so, it is crucially important to take into consideration not only the impact of a particular type of physical activity, but also a broad range of forms and severity of disabilities, as well as their various etiologies and origins of health limitations [2,3,4,5,6,7,8,9,10,11].

Basically, it is known that congenital disabilities have mainly genetic origins and are usually associated with gene mutations responsible for collagen synthesis, resulting in connective tissue dysplasia (CTD) manifestation. In accordance with data from epidemiologic studies, CTD occurrence correlates with high anthropogenic load in some regions [12,13,14,15,16,17,18,19]. Moreover, CTD phenotypic markers reveal themselves in most parts of human systems and organs, including the cardiovascular system, particularly in the appearance of additional structures as false tendons in the left ventricle (FTLVs).

A number of studies on the significance and clinical value of these structures are reported [19,20,21,22,23,24,25,26,27,28,29,30,31,32,33,34,35,36,37,38,39,40,41,42]. In our previous studies, we focused on the estimation of the impact of FTLVs on cardiac function in healthy athletes [43,44,45]. Our findings showed significant interrelations between the presence of anatomical anomalies in LV structure and myocardium regional function, in particular, the increase in mechanical asynchrony in LV wall motion [44,45,46,47].

In general, the peculiarities of cardiac regional structure and function determine heart functional reserve, i.e., its ability to maintain adequate pump function in the face of increasing load to the myocardium [46]. Therefore, LV anomalies may contribute the additional effect on cardiac response to exercise loads in healthy subjects and people with disabilities. This issue remains not well studied in individuals with disabilities engaged in athletic activity.

In the present work, we investigated a large group of subjects with disabilities, CTD, and LVFTs from the different types of nosology and adaptive sports disciplines to check for general patterns of cardiac response to physical activity.

The extent to which the type of sports or the type of disability determines the involvement of the heart functional reserve is the main aim of the proposed research. Meanwhile, the fundamental point of this study is to test the hypothesis that, regardless of the origin of disability, type of sport, gender, or age of athletes, false tendons in the LV determine the heart functional reserve.

## 2. Materials and Methods

### 2.1. Study Design and Participants

The study was conducted in the research Laboratory “Functional testing and complex control in sports” of Institute of Physical Education, Sports and Youth Policy, Ural Federal University (Yekaterinburg, Russia) in collaboration with Center of Adaptive Sports “Rodnik” of Sverdlovsk Regional Ministry of Sports and Regional Federations and organizations of Sport for the Disabled from 2023 to 2025.

Six hundred and ten individuals with various forms and severity of disabilities were recruited for the study. Participants were aged from 6 to 60 years. All studied subjects were grouped in reference to type of disability and sports specialization. This enabled us to treat this cohort as analytically comparative. The distribution of participants was as follows: 47 athletes with hearing impairment (HI) engaged in cross-country skiing (n = 21), swimming (n = 18), and sports orienting (n = 8); 86 athletes with intellectual and developmental disabilities (IDDs) training in equestrian sport (n = 65) and football (n = 21); 110 athletes with vision impairment (VI)—marathon runners (n = 23), swimmers (n = 62), and skiers (n = 25); 367 athletes with musculoskeletal and physical impairments (MPI)—cross-country skiers (n = 48), para-hockey-players (n = 24), para-football players (n = 36), swimmers (n = 67), para-equestrians (n = 45), para-tennis players (n = 15), wheelchair dancers (n = 27), and beginners in sports, practicing different sports activities (n = 146).

Before complex testing of cardiovascular screening, each participant was comprehensively instructed on purpose, testing protocol, investigation details, and safety measures. In all cases, athletes were recommended to inform the research team about any discomfort or complaints that appeared during testing procedures. Laboratory staff followed appropriate international guidelines while performing overall tests and measurements. Informed consent was obtained from all subjects or their parents/official guardians (in cases with participants under 18 years) involved in the study.

### 2.2. Cardiology Screening

In terms of the current purpose of the study, the choice of methods included body composition and anthropometric measurements, resting 12-leads ECG, hemodynamic monitoring, transthoracic echocardiography (TTE), exercise testing, and further statistical analysis. Before comprehensive cardiology screening, each participant completed a detailed medical history questionnaire. All cardiologic examinations were performed in accordance with international guidelines [48,49,50,51,52,53,54,55,56,57,58,59,60,61,62,63,64] by the same operators and supervised by a specialist in sports cardiology and functional diagnostics.

### 2.3. Body Composition Analysis

Anthropometry and body mass distribution was measured with the use of a Multi Frequency Segmental Body Composition Analyzer MC-980 (TANITA Corporation, Tokio, Japan). The following parameters were measured and further analyzed: body mass (BM, kg), muscle mass (MM, kg and %), fat tree body mass (FFM, kg and %), and body mass index (BMI).

### 2.4. Twelve-Leads Resting ECG

Twelve-leads ECG, a basic non-invasive informative method, was performed in accordance with international standards and guidelines [49,50,51] with the use of a CARDIOVIT AT-104 PC diagnostic system (Schiller AG, CH-6341 Baar, Switzerland) with further manual remeasurement and interpretation in each case. The following ECG variables were analyzed: HR, electric heart axis position, duration and amplitude of P, Q, R, S, T waves, duration of PQ, QT and corrected QT (QTc) intervals, and QRS complex. To avoid potential errors in automatic measurements, all variables were remeasured by manual detection of the beginning and the end of ECG waves in II or V5 lead. The end of the T-wave was identified by the threshold method. Formulas applied for QT interval correction were chosen in line with calculated HR. Bazett’s formula (QTc = QT/√(RR)), as the most commonly used, was chosen for HR range from 60 to 100 bpm. In cases with HR lower than 60 bpm or higher than 100 bpm, the Framingham formula was applied (QTc = QT + 0.154 × (1 − RR)). In terms of athletic background of the studied subjects, voltage criteria were excluded from consideration. Special attention was drawn to both rhythm and conductivity disorders, as well as criteria of myocardium electrical instability, frequently considered as risk factors of sudden cardiac arrest in athletes under the impact of training and competitive activities. The main focus was placed on markers of athletic physiologic changes according to European Guidelines (ESC 2018) and juvenile ECG patterns—attributes of growing hearts in kids and adolescents. Resting ECG evaluation enabled us to estimate long-term cardiac response to exercise loads and, in some cases, its deviation from normal values.

### 2.5. Transthoracic Echocardiography (TTE)

TTE was performed with the use of a “Samsung HM70 EVO” diagnostic ultrasound system (SAMSUNG MEDISON Co., Ltd., Seoul, Republic of Korea). The quantification of global LV structure and function was undertaken in M-mode and B-mode from parasternal and apical views in accordance with ACC/AHA Guidelines for the Clinical Application of Echocardiography 2012 [52]. LV structural measurements (LV size and mass) and functional parameters (LV global systolic and LV diastolic function) were performed. LV endocardial image at the end of diastole and the end of systole were chosen by means of synchronization of echo- and ECG signals. The biplane Simpson method (four chamber and two chamber [4C + 2C]) was applied for LV chamber quantification [53].

The following LV parameters, as reliable indicators of cardiac contractile and pumping functions as well as long-term adaptation to exercise activity, were estimated: LV volumes (end-diastolic volume (EDV, mL) and end-systolic volume (ESV, mL)), stroke volume (SV, mL), and ejection fraction (EF, %). Indexing of TTE measurements was excluded due to specifics of the studied group, including individuals with musculoskeletal disorders, amputees, persons with cerebral palsy, and wheelchair athletes. From this point of view, indexing cardiac variables to body-surface area (BSA) could potentially cause an error in interpreting results. The significance of LVFTs topology in terms of LV mechanical asynchrony increase and LV functional reserve impairment was established in our previous studies [43,44]. Thus, only transverse and oblique LVFTs mainly located at basal and/or medial portions of the LV chamber were considered.

### 2.6. Hemodynamic Monitoring

Hemodynamic monitoring was performed with the use of a MARG 10-01 impedance cardiography device (Microlux, Chelyabinsk, Russia) in active orthotest. Investigation included estimation of the current cardiac functional state of participants and comparison of the obtained hemodynamic data with normal values [54].

The following hemodynamic indicators were recorded and further analyzed in supine and upright body position: blood pressure (BP, mm Hg), heart rate (HR, bpm), stroke volume (SV, mL), stroke index (SI, mL/m^2^), cardiac index (CI), end-diastolic volume (EDV, mL), end-diastolic index (EDI, mL/m^2^), and ejection fraction (EF, %). SI and EDI were calculated as the ratio of SV and EDV to the body surface area BSA (SV/BSA). The inability to perform orthotest in wheelchair participants (n = 60) was the reason to undertake hemodynamic measurements only in supine position in these cases.

### 2.7. Exercise Testing

Exercise performance and cardiovascular adaptation to exercise loads data were collected during exercise testing (ET) with ECG monitoring [55]. The types of ergometers and test protocols used were carefully selected in reference to the requirements of the exact kind of sport and verified to each case (type and severity of disability of the participants). A range of methods were modified to coincide with individual functional abilities. In contemporary sports medicine and sports practice, maximal stress tests are supposed to carry out exercise activity till exhaustion until maximal oxygen consumption (VO_2_max) criteria are achieved and subjective signs and symptoms are observed. VO_2_max is characterized by maximum work of the oxygen-transport system. Maximum relative oxygen consumption (VO_2_max, mL·kg^−1^·min^−1^) is an integral parameter which serves as a reliable indicator of heart functional reserve and aerobic performance [56,57,58,59]. Considering a wide range of participants, VO_2_max was measured by means of both direct method (n = 466) utilizing a gas-exchange analyzer Fitmate PRO (Cosmed S.r.l., Albano Laziale—RM, Italy) [60], as well as indirect calculation (n = 144) of this parameter using the ACSM equation [61,62,63], based on attained load (watt) or speed (km·h^−1^) and athletes’ anthropometric parameters in cases with special restrictions.

Additionally, the appropriate selection of ergometer required an individual approach and was strongly related to the physical abilities of participants. Although overall subjects underwent the maximal incremental RAMP test (ACC/AHA 2002 Guidelines update for exercise testing, 2006), the type of ergometer varied within the studied group. But to obtain clear and accurate data, the test design was similar in the subgroups formed according to type of disability and sports specialization.

Young athletes aged under 12 years (n = 183) underwent the step-test with ECG monitoring. The modified Harvard step-test protocol was selected [65] with lower height of the step.

The cycle exercise test was conducted with the use of a Schiller stress-testing system (Schiller AG, CH-6341 Baar, Switzerland) and a portable gas-exchange analyzer. The RAMP protocol was applied with load increment (watt/min) equal to ½ body mass starting from 0 watts at the warm-up stage. It was recommended to keep pedaling at 80 rpm during the whole test. The test was terminated when athletes could not keep the required cadence or symptoms of exhaustion appeared [65]. The following parameters were estimated: HR (bpm), VO_2_ (mL·kg^−1^·min^−1^), power (P, watt), breathing rate (Rf, 1·min^−1^), minute ventilation (VE, l·min^−1^), and maximal values of these indices. All variables were recorded throughout the whole test and further analyzed. Ventilatory thresholds, both 1st (VT_1_) and 2nd (VT_2_), were evaluated after test termination.

An exercise test (ET) with the use of an arm-ergometer Technogym Top Bike (TECHNOGYM S.p.A, Cesena (FC), Italy) was applied for wheelchair participants and those with functional limitations in lower extremities with normal capabilities in upper extremities (n = 37). The same physiological parameters as described above were evaluated during arm cranking ET.

### 2.8. Statistical Analysis

Descriptive statistics were calculated for all physiological parameters, including the mean (M), standard deviation (SD), and the minimum and maximum observed values. Prior to conducting comparative analyses, the dataset underwent a multi-stage preprocessing pipeline that included variable type harmonization, handling of missing data, and verification of measurement quality. Given the high inter-individual variability characteristic of athletes with health impairments, data filtering was performed as conservatively as possible, with a strong emphasis on preserving genuine biological variability.

To identify clearly artefactual values, within-group Z-normalization was applied across age strata, followed by exclusion of observations exceeding |z| > 3. This approach can be interpreted as intra-group confidence interval filtering (approximately μ ± 3σ), ensuring retention of physiologically plausible values within each age category.

The normality of each variable was assessed using the Shapiro–Wilk test, and homogeneity of variances across groups was evaluated using Levene’s test. When the distribution of a variable substantially deviated from normality and/or group variances were heterogeneous, comparisons across nosology categories and sport-type groups were performed using the Kruskal-Wallis H-test, a non-parametric analogue of one-way ANOVA. For variables that satisfied the assumptions of normality and homoscedasticity, one-way ANOVA was applied, with effect size estimated via eta-squared (η^2^), representing the proportion of variance in the dependent variable explained by the grouping factor (sport type or nosology). In applied sports cardiology, a “medium” effect size (e.g., η^2^ around 0.06) denotes a practically significant, marked difference in outcomes (like heart adaptation or training response) from an intervention, while a “small” effect size (e.g., η^2^ around 0.01) indicates a statistically significant but clinically less important, minor change, frequently requiring larger groups or longer studies to identify reliably or justify major changes in practice. For non-parametric comparisons (Kruskal-Wallis), effect size was calculated using epsilon-squared (ε^2^), which serves as a rank-based analogue of η^2^. When the global test (ANOVA or Kruskal-Wallis) indicated statistically significant differences (*p* < 0.05), post hoc pairwise comparisons were conducted using Tukey’s HSD (for ANOVA) or Mann–Whitney U-tests with Holm correction (for non-parametric pairwise contrasts).

We used a data preprocessing algorithm: automatic detection of the age column and formation of five-year age groups; selection of all numerical variables, excluding age and the identifier; calculation of means and standard deviations for each numerical variable separately within each age group; computation of within-group z-scores and replacement of values with |z| > 3.0 by missing values (NaN) at the level of individual cells, without deleting rows (participants); saving the cleaned dataset to a separate file with the specified original path and name.

First, filtering was performed not at the patient level but at the level of individual measurements: values exceeding |z| > 3.0 were replaced by missing values only in the corresponding cells, without deleting rows. This reduces the influence of single measurement artefacts or input errors on group estimates but does not exclude a participant entirely from the analysis for other variables.

Second, z-scores were calculated within five-year age groups, which takes into account the age dependence of physiological variables and makes the |z| > 3.0 criterion more clinically reasonable: values differing by more than three standard deviations from the mean for their age subgroup, in a sports population with prior medical screening, are highly likely to represent measurement artefacts or incorrect input. The threshold of 3.0 standard deviations is traditionally considered conservative and, under distributions close to normal, affects less than 0.3% of values.

All stages of data preprocessing, statistical analysis, and effect size estimation (η^2^ and ε^2^) were implemented in Python 3.13.3 (Python Software Foundation, Wilmington, DE, USA) using a modern scientific computing stack that included the libraries pandas, numpy, scipy.stats, and statsmodels, as well as the visualization tools matplotlib and seaborn. The analytical pipeline was executed in a Python 3.13.3 environment, with the following versions of key libraries: pandas 2.2.3, numpy 2.2.6, scipy 1.16.0, statsmodels 0.14.5, matplotlib 3.10.3, and seaborn 0.13.2. These versions ensure full reproducibility of all computational procedures and conform to contemporary standards of computational statistics.

The use of this software environment enabled standardized procedures for data loading, filtering, and transformation, the correct application of both parametric and non-parametric group comparison methods, the computation of effect sizes, and the generation of visualizations essential for the interpretation of between-group differences. This approach is consistent with the best international practices in computational research and ensures both transparency and reproducibility of the results.

## 3. Results

### 3.1. Echocardiographic Markers of CTD and TTE Data

**Figure 1:**
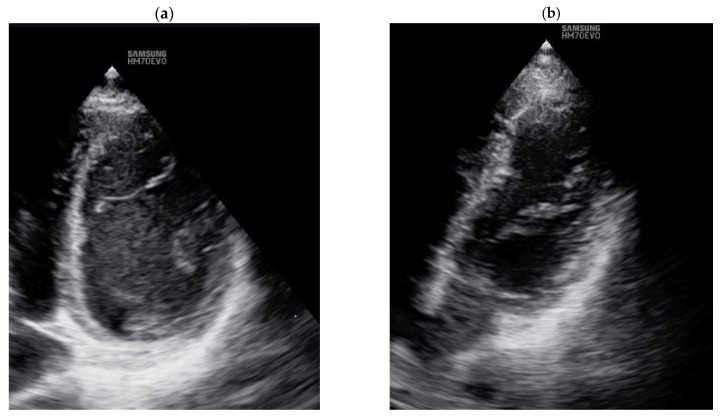
Examples of TTE LV visualization with false tendons. (**a**) Transverse LVFT in the low median portion of LV cavity, connecting interventricular septum and free LV wall. Two LVFTs are partially visible from the side of free LV wall. (**b**) Two thick transverse LVFTs partially visible in the medium portion of the LV cavity, one transverse LVFT located in the low median LV portion partially visible from the side of free LV wall.

TTE screening revealed the presence of CTD markers in the LV in overall studied disabled individuals despite the type or severity of impairment. Additional connective tissue structures, from 2 to 5 (3.4 ± 1) false tendons in the heart left ventricle (LVFTs), were found in 100% of examined individuals (Figure 1). Notably, we considered only significant LVFTs: thick (>2 mm) transverse and/or oblique LVFTs, connecting opposite LV walls, attached to interventricular septum and free LV wall predominantly located at basal and/or medial portions of the LV chamber.

TTE data of measured LV parameters were as follows: EDV—76.62 ± 23.17 (40–133) mL, ESV—30.32 ± 11.83 (15–66) mL, SV—43 ± 15.33 (19–77) mL, EF—57.17 ± 7.4 (40–67)%. These results show that all major indicators varied widely.

### 3.2. Twelve-Leads Resting ECG Findings

Twelve-leads ECG implication and further interpretation made it possible to identify common features in heart electrical activity within the whole group of studied subjects. Mean values of the measured parameters (M ± SD (min–max)) were as follows: duration of corrected QTc interval—417.3 ± 26.8 (334–524) ms, PQ interval—142.1 ± 20 (90–230) ms, QRS complex duration—93.3 ± 10.8 (68–136) ms. Although the findings described above demonstrate a wide range of variables values, our attention was drawn to higher-than-normal values in the general population duration of QTc interval [49,50]. Basically, this parameter characterizes electrical LV activity (repolarization and depolarization) and is closely related to LV functioning. Moreover, pronounced prolonging of this interval serves as a risk factor of life-threatening arrythmias and sudden cardiac arrest during exercise activity [48,49,50]. The data obtained showed that less than 1% of studied subjects had pronounced prolongation of QTc interval (n = 3). Considering absence of clinical symptoms and family history, these participants were advised to further undergo in-depth cardiovascular examination.

### 3.3. Hemodynamic Monitoring

Hemodynamic monitoring revealed decreases in most of the analyzed variables in comparison to normal values in the healthy population. We found that in the supine position, the majority of hemodynamic indices varied widely and were lower than predicted. EDI value was 80.6 ± 14.6 (42–118) mL/m^2^, EF—62.03 ± 3.6 (43–76)%, SV—80.8 ± 23.3 (23–135) mL. Notably, in upright position (orthotest), indices values significantly changed—on average, EDI decreased by 16.2 ± 10.4 and variability was excessive from 1.1 to 63.0%. EDI value in orthostasis was 67.8 ± 14.3 mL/m^2^, while EF—62.03 ± 3.6 (43–76)%.

### 3.4. Exercise Testing

Effective cardiovascular adaptation to exercise loads is vital and one of the most important requirements for optimizing para and athletic performance. Sports for disabled people is not an exception, as this category of population engaged in sports activity experiences similar training and competitive activity as healthy athletes. Analysis of data obtained during ET showed controversary patterns in the studied group: (i) VO_2_max, an integral index of exercise performance, varied widely and confirmed the facts described above regarding wide variations of values from 17.9 to 75.5 mL·kg^−1^·min^−1^ within the whole group, although the mean value was 46.5 ± 10 mL·kg^−1^·min^−1^; (ii) maximum HR was lower than predicted (168.2 ± 42.3 bpm) and ranged from 127 to 222 bpm, which points to different levels of cardiac response irrespective of type of disability or sports specialization, though lowered aerobic abilities on average. These findings demonstrate the need for further in-depth analysis to explain these patterns of cardiovascular adaptation.

### 3.5. Between-Group Analysis

#### 3.5.1. Differences Across Sport Types

After data cleaning and outlier removal, the final dataset comprised 581 observations and 141 variables. Differences in VO_2_max and QTc across sport types and nosological groups were analyzed using one-way ANOVA or the Kruskal-Wallis test, depending on whether the assumptions of normality and homogeneity of variance were met. Notably, statistically significant findings with small effect sizes were not interpreted as clinically meaningful and needed further validation.

**Figure 2:**
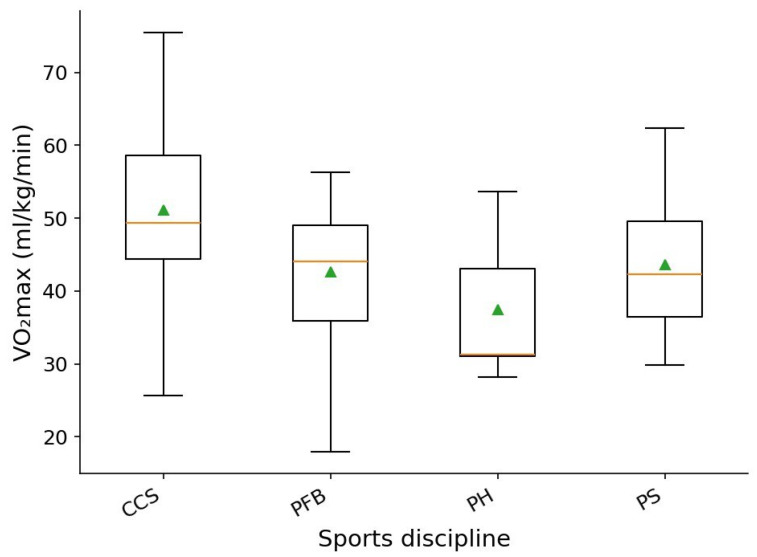
Distribution of VO_2_max across sports disciplines. Boxes show the interquartile range; the horizontal line inside each box marks the median and triangles indicate the mean. PS—para-swimming, CCS—cross-country skiing, PH—para-hockey, PFB—para-football.

The analysis revealed statistically significant differences in VO_2_max across athletic disciplines (one-way ANOVA, *p* = 0.00063; η^2^ = 0.1577), corresponding to a medium-sized effect. This indicates that sport type is a meaningful determinant of variation in aerobic performance. These findings are visually supported by the VO_2_max by sport chart (Figure 2), where distributions across sport groups show clear heterogeneity in both median values and variability ranges.

Athletes participating in cross-country skiing demonstrated the highest aerobic capacity, with a median VO_2_max of approximately 50 mL·kg^−1^·min^−1^, an interquartile range of roughly 45–58 mL·kg^−1^·min^−1^, and maximum individual values reaching 75 mL·kg^−1^·min^−1^. This reflects the expected high level of aerobic conditioning in this endurance-oriented sport.

Para-football players exhibited median VO_2_max values around 44–45 mL·kg^−1^·min^−1^, accompanied by a wide performance range—from 18 to 56 mL·kg^−1^·min^−1^—highlighting substantial within-group variability.

Para-swimmers showed median VO_2_max values of approximately 42–43 mL·kg^−1^·min^−1^, with an interquartile range of 36–49 mL·kg^−1^·min^−1^, indicating a moderately developed level of aerobic fitness.

The lowest VO_2_max values were observed in para-hockey players, who demonstrated a median of 31–32 mL·kg^−1^·min^−1^, an interquartile range of 30–43 mL·kg^−1^·min^−1^, and maximum values not exceeding 54 mL·kg^−1^·min^−1^.

**Figure 3:**
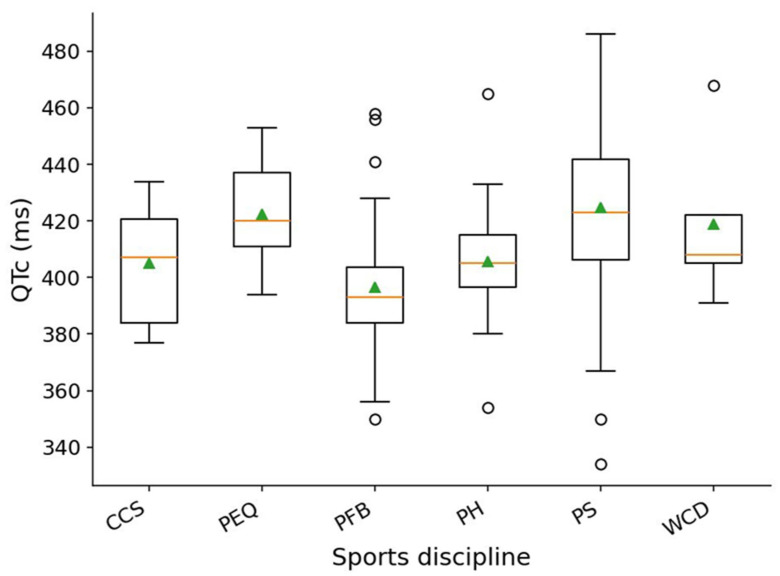
Distribution of QTc across sports disciplines. Boxes show the interquartile range; the horizontal line inside each box marks the median and triangles indicate the mean, while open circles indicate outliers. PS—para-swimming, CCS—cross-country skiing, PEQ—para-equestrian, PH—para-hockey, PFB—para-football, WCD—wheelchair dancing.

The QTc interval also showed statistically significant differences across sport disciplines (Figure 3). As the QTc distribution violated both normality (Shapiro–Wilk test) and homogeneity of variances (Levene’s test), between-group comparisons were conducted using the non-parametric Kruskal-Wallis H-test.

The results were significant (*p* < 0.00001), and the effect size calculated using the epsilon-squared statistic was ε^2^ = 0.1462, corresponding to a medium effect. This indicates that sport type exerts a meaningful influence on the variability of ventricular repolarization.

The QTc by sport plot illustrates the distribution of QTc values across disciplines, and several pronounced between-group differences can be observed.

Athletes engaged in para-swimming exhibited median QTc values around 420–425 ms, with a notably widespread range from approximately 335 ms to 485 ms, making this discipline one of the most heterogeneous in terms of QTc variability.

Para-skiers demonstrated lower median values—approximately 405–410 ms—with substantially narrower dispersion, reflecting a more homogeneous profile of cardiac electrical activity.

In para-equestrian athletes, QTc values were higher again, with median values near 420 ms and upper-quartile values reaching approximately 450 ms. This profile resembles that of para-swimmers, though with fewer extreme outliers.

Para-hockey players showed median QTc values around 405 ms, with moderate variability and a comparatively narrower range than in other disciplines.

Para-football players demonstrated one of the lowest median QTc intervals—approximately 390–395 ms—combined with a wide overall range (350 to 430 ms), suggesting pronounced within-group heterogeneity.

Wheelchair dancers displayed median QTc values of about 408–410 ms, with a moderate spread: lower values around 390 ms and upper values approaching 470 ms.

Taken together, the visualization indicates heterogeneous QTc variability between sport types, expressed in differences in median values, interquartile ranges, and the presence of outliers. These visual patterns align well with the Kruskal-Wallis test results, reinforcing the presence of systematic between-group differences.

#### 3.5.2. Differences Across Nosology Groups

When comparing nosology categories, statistically significant differences in VO_2_max were identified (one-way ANOVA, *p* = 0.01028; η^2^ = 0.0717). The effect size corresponds to a small effect (η^2^ = 0.07), indicating moderate but reproducible differences in aerobic capacity across participants with different types of health conditions.

For VO_2_max and QTc comparisons, only those sports and nosology categories were included in the analysis, in which, after preprocessing the data, there were at least five valid observations for the corresponding variable. Groups with fewer measurements were excluded from the intergroup comparison and, accordingly, are not represented in the figures.

**Figure 4:**
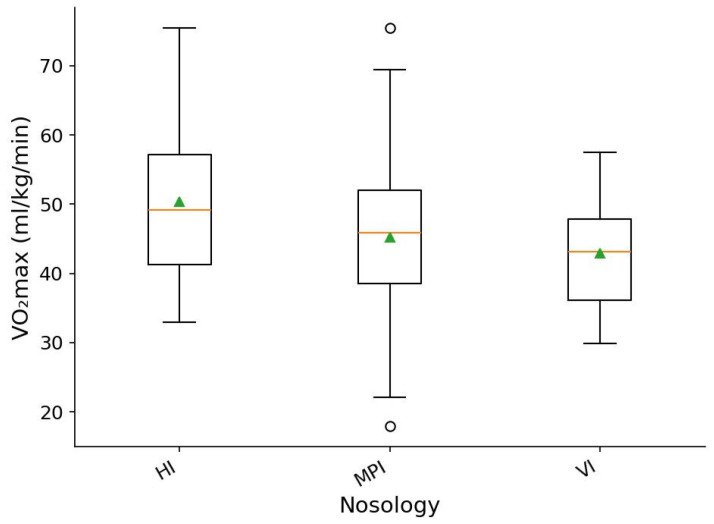
Distribution of VO_2_max across disability types. Boxes show the interquartile range; the horizontal line inside each box marks the median and triangles indicate the mean. HI—hearing impairment, VI—vision impairment, MPI—musculoskeletal and physical impairments; open circles indicate outliers.

As illustrated in Figure 4, the between-group contrasts are less pronounced than those observed across sport types, yet they follow a discernible pattern.

Participants with hearing impairments demonstrated the highest VO_2_max levels, with median values around 49–50 mL·kg^−1^·min^−1^. The interquartile range was relatively broad—from approximately 41 to 56 mL·kg^−1^·min^−1^—and maximum values reached up to 75 mL·kg^−1^·min^−1^, indicating substantial within-group variability.

Individuals with vision impairments exhibited lower VO_2_max levels, with a median near 43 mL·kg^−1^·min^−1^ and a narrower interquartile range of approximately 36–48 mL·kg^−1^·min^−1^. This suggests a more homogeneous level of aerobic fitness within this group.

Participants with musculoskeletal impairments showed intermediate median VO_2_max values of about 46 mL·kg^−1^·min^−1^; however, their distribution was more heterogeneous, spanning from isolated low values (18–22 mL·kg^−1^·min^−1^) to high ones (70–75 mL·kg^−1^·min^−1^). This pattern reflects substantial physiological heterogeneity in this category.

Taken together, although the effect size remains small, both the ANOVA results and visual inspection confirm the presence of moderate differences in aerobic capacity across nosology groups. These differences, however, are notably smaller than those associated with sport type, which is consistent with the expected role of training modality as a potentially stronger determinant of VO_2_max than clinical classification alone.

When analyzing QTc intervals across nosology categories, statistically significant differences were identified (Kruskal-Wallis, *p* = 0.02185; ε^2^ = 0.0175). The effect size (ε^2^) was very small, indicating a weak but statistically detectable tendency toward variation in ventricular electrical activity among participants with different health conditions.

**Figure 5:**
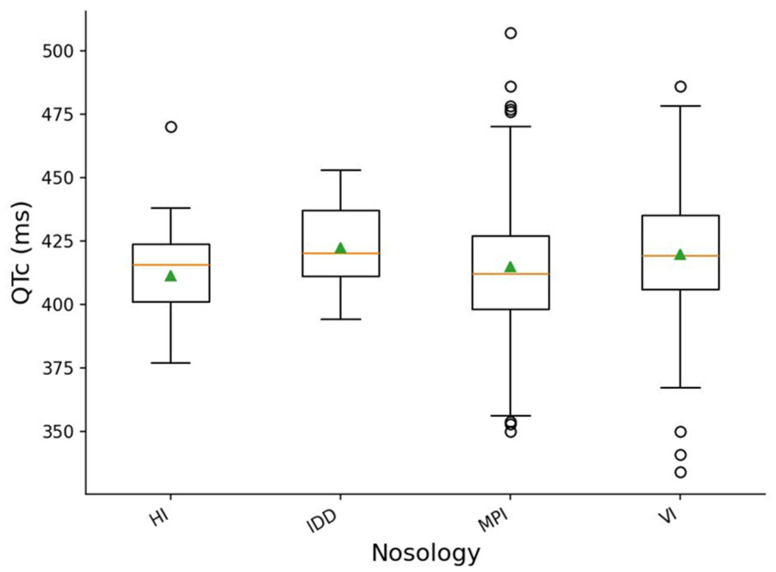
Distribution of QTc across disability types. Boxes show the interquartile range; the horizontal line inside each box marks the median and triangles indicate the mean. HI—hearing impairment, IDD—intellectual and developmental disabilities, VI—vision impairment, MPI—musculoskeletal and physical impairments; open circles indicate outliers.

The graphical representation of QTc distribution across disability types (Figure 5) shows that the distributions largely overlap across groups, yet several characteristic differences in median positions and variability remain observable.

Participants with hearing impairments demonstrated median QTc values around 415–420 ms with moderate variability, ranging from approximately 400 to 430 ms, along with occasional low values near 380 ms.

In the group with intellectual disabilities, the median QTc was slightly higher (420–422 ms), and the interquartile range was shifted toward higher values relative to the hearing impairment group.

Participants with vision impairments exhibited the largest spread of QTc values: the median was approximately 420–423 ms, but the variability extended widely—from around 405 ms to 480 ms—and several low outliers (335–360 ms) were also present. This pattern reflects substantial within-group heterogeneity.

Finally, individuals with musculoskeletal impairments showed median QTc values in the range of 410–415 ms, with moderate dispersion that included occasional high values (470–510 ms), further indicating physiological heterogeneity within this category.

Although the effect size was small, the results of the Kruskal-Wallis test and the visual inspection of the plot confirm the presence of moderate differences in QTc across nosology groups, despite extensive overlap of distributions. This aligns with the broader physiological variability characteristic of populations with diverse health conditions and underscores the need for further investigation into potential clinical and training-related factors influencing ventricular repolarization in these athlete groups.

#### 3.5.3. Effect Sizes

**Figure 6:**
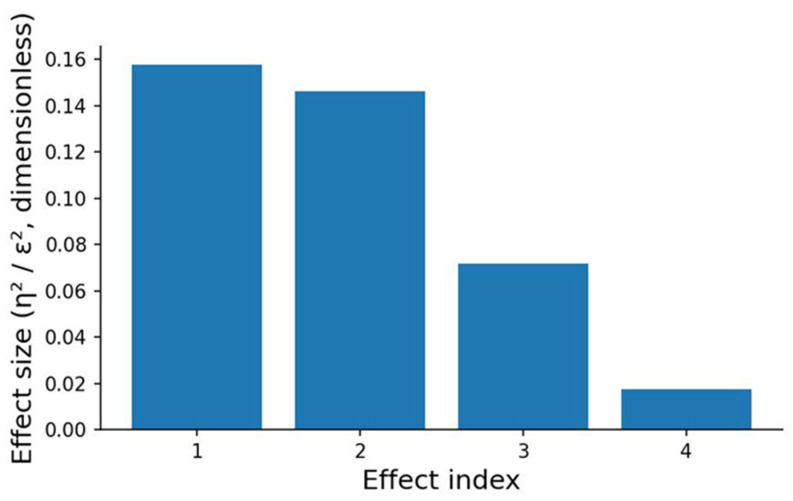
Effect sizes (η^2^/ε^2^) for the associations between grouping factors and outcome variables. Bars show the proportion of variance in VO_2_max (mL·kg^−1^·min^−1^) or QTc (ms) explained by sports discipline or nosology. Effect index: 1—sports discipline and VO_2_max (η^2^); 2—sports discipline and QTc (ε^2^); 3—nosology and VO_2_max (η^2^); 4—nosology and QTc (ε^2^).

An integrated comparison of effect sizes is presented in Figure 6. The plot displays four calculated effect sizes (η^2^ or ε^2^), corresponding to the influence of two factors—sport type and nosology—on VO_2_max and QTc. Each bar represents the proportion of between-group variance explained by a given factor.

The strongest effect was observed for sport type in relation to VO_2_max, with an effect size of η^2^ = 0.158, which corresponds to a medium effect. This indicates substantial differences in aerobic capacity across sporting disciplines and underscores the central role of sport-specific training stimuli in shaping cardiorespiratory adaptation.

A comparable effect magnitude was found for sport type in relation to QTc (ε^2^ = 0.146). Although QTc reflects ventricular repolarization—an indicator less directly linked to training volume—its variation across sport types may reflect differences in habitual training load, sympathetic activation profiles, and discipline-specific patterns of cardiac remodeling.

In contrast, the influence of nosology on VO_2_max was markedly weaker (η^2^ = 0.072), representing a small effect. This suggests that health-related functional limitations exert only a modest impact on aerobic performance when compared with the influence of sport specialization.

The smallest effect was observed for nosology in relation to QTc (ε^2^ = 0.018). This value falls within the range of very small effects, indicating a weak, though statistically detectable, tendency toward variation in QTc across health-related categories.

Taken together, the diagram demonstrates that sport type is a substantially stronger determinant of physiological variability than nosology classification. This result highlights the dominant role of sport-specific training exposure and athletic conditioning in shaping both functional (VO_2_max) and electrophysiological (QTc) characteristics of the heart among athletes.

## 4. Discussion

Although the “First global physical activity and sedentary behavior guidelines for people living with Disability” was published a few years ago (2021) [1], the significance of the study is potentially low in relation to athletes with disabilities. The reasons for the undertaken decision are rather clear: (i) issues with forming homogeneous groups by age, gender, nosology, functional class, and sports specialization; (ii) lack of data concerning interrelations between congenital or acquired disability and systemic adaptation features to exercise loads of various types and intensity. Meanwhile, the importance of these studies should not be underestimated and should gain notable attention due to not only the progressive growth in disability worldwide, but also the significant increase in unspecified etiology in the overall population.

Notably, connective tissue dysplasia has become widespread within previous decades [12]. It should be emphasized that CTD is highly related to a range of disabilities. A number of studies demonstrate the interrelation between congenital impairment of vision, hearing, musculoskeletal system, and collagen synthesis disorders [15,16,17,18]. Some authors claim coexistence of different types of vision impairment and musculoskeletal system in subjects with non-differentiated CTD forms [17,18,66,67]. Along with that, there are also data on extremely high (up to 70%) prevalence of synthesis collagen impairment in the embryonic stage in persons with congenital hearing loss [17,68]. According to our previous studies, the occurrence of CTD in various age groups may come up to 100% [65].

CTD phenomenon is also related to anthropogenic transformation of the environment. Based on epidemiologic data, in some regions, the CTD occurrence may come up to 70–90% [12,19,37,41]. Particularly, in the Urals’ region (Russia), the prevalence of CTD is extremely high [43,44]. Regarding the fact that participants in the present study were predominantly representatives of this region, it was not a surprise that this cohort has manifestations of CTD as well. In actuality, performed screening tests confirmed CTD signs in all studied subjects. To document the presence of CTD, all participants underwent TTE for the identification of LVFTs, which are considered as strong CTD markers.

In general, CTD is a micro-evolutional phenomenon related to congenital collagen synthesis impairment, resulting in the decrease in connective tissue stiffness [46]. On the systemic level, CTD development results in the appearance of additional connective tissue structures in hollow organs, increasing structural integrity. This phenomenon is similar to how the strength of crane structures or bridge spans is increased by enhancing the rigid lintels between parts.

In the heart, with CTD presence, multiple additional tendons appear, covering the endocardium, yet these are hardly visualized by means of TTE. Additionally, massive FTs appear, mostly in the LV, connecting extensive parts of the LV chamber walls. These structures are well visualized, and their number may come up to 5–7 units per LV, depending on elastic properties of cardiac wall connective tissue. It was found that all studied subjects had more than two significant FTs per LV regardless of type of disability, sports specialization, gender, or age.

In fact, this finding was not surprising, as previous studies in healthy young athletes revealed similar TTE results [43,44,45]. Contemporary cardiology does not consider the LVFTs phenomenon as a pathological finding, as heart function at rest remains within the normal values, as in healthy subjects without CTD phenotypic markers. In actuality, the obtained results show that mostly cardiorespiratory function parameters in the studied subjects varied within the normal values irrespective of type of sport or nosology. At the same time, cardiac response to exercise loads enabled us to reveal the differences across the studied group. This was manifested primally in the inability to complete exercise tests at the required heart rate as well as excessive spread of VO_2_max values. These findings confirm the different extent of heart functional reserve involvement in increasing load depending on both type of sport and nosology. Along with this, effects of types of sports had priority over type of disability.

It seems evident that general principles and mechanisms of cardiovascular adaptation must be similar in healthy athletes and those with disabilities, and the main mobilization sources of heart reserve and their activation during exercise activity are well studied. At the same time, the present study was undertaken in athletes with disabilities, CTD, and LVFTs. This circumstance distinguishes our work from others.

According to our previous research findings in athletes with CTD, the appearance of FTs in the LV cavity was associated with an unusual level of mechanical asynchrony of LV wall motion [44,45,46,47]. In particular, it was shown that myocardium electrical and mechanical inhomogeneity was significantly higher in athletes with CTD in comparison to healthy ones [43,44,45]. In addition, the physiological value of adaptation, expressed in involvement of significantly higher inotropic and coronary heart reserves per unit of load, was also observed [43]. These insights support the notion that CTD is an important determinant of heart functional reserve and adaptation to exercise loads.

Data from statistical analysis on significant impact of athletes’ kind of sport or nosology on VO_2_max value enabled us to hypothesize the impact of LVFTs on the obtained result. Qualitatively, it coincides with our earlier studies in healthy athletes [43,44]. Additionally, our hypothesis was supported by the results of the resting ECG evaluation, where the duration of QTc interval was longer, as in healthy subjects with CTD [43]. LVFTs may affect excitation spread throughout LV walls due to the presence of the heart conductive system cells in these structures [38].

In summary, the present study focused on the fundamental bases of cardiovascular adaptation in disabled individuals with CTD, and on the future development of recommendations for safe training prescriptions in this category of population. From our viewpoint, the results obtained have principal importance and should be considered in medical support of training processes in athletes with disabilities and CTD. It is recommended to complete individualized comprehensive preparticipation cardiovascular screening in athletes with disabilities, including TTE and exercise testing with 12-leads ECG recording, for cardiac risk prevention.

### Limitations

This study has several limitations. Initially, the design of the present research was cross-sectional. The extremely high prevalence of CTD in the region where the study was conducted explains the absence of a non-CTD comparison group of athletes with disabilities. Additionally, heterogeneity of age, training background, and testing protocols, and lack of longitudinal validation limit the significance of the results obtained. Mixing direct and indirect VO_2_max assessments may serve as a potential source of measurement variability. On the other hand, statistical analysis allowed us to minimize the error, yet not to completely exclude it. Thus, the reliability of the presented data should be investigated further in larger and less heterogeneous groups by means of the same methods in all subjects.

## 5. Conclusions

Heart functional capacities were evaluated in a large group of individuals with disabilities engaged in various sports—para-swimming, para-football, para-hockey, paraskiing, para-athletics, para-tennis, etc. All studied subjects had CTD confirmed with the use of TTE by presence of thick transverse LVFTs. The obtained results of the undertaken study demonstrate that in athletes with disabilities and CTD, sports specialization involves the heart functional reserve to a greater extent than the type of disability. To prescribe training loads for people with disabilities and CTD, individual cardiology screening with an emphasis on VO_2_max and QTc is necessary.

## Data Availability

The data presented in this study are available on request from the corresponding author due to privacy and ethical restrictions.

## Abbreviations

The following abbreviations are used in this manuscript:

LV	Left ventricle
CTD	Connective tissue dysplasia
LVFTs	False tendons in the LV
TTE	Transthoracic echocardiography
ECG	Electrocardiography
VO_2_max	Maximum oxygen consumption
ICG	Impedance cardiography
QTc	Corrected QT interval
ET	Exercise testing
